# College and Hospital Notes

**Published:** 1904-11

**Authors:** 


### COLLEGE AND HOSPITAL NOTES.

The board of managers of the new Mercy Hospital has appointed the following-named physicians as the attending staff: medical department, James W. Nash, James E. Culbert, A. L. Benedict; alternates, Ira F. Trevett, James J. Brown, Robert E. DeCeu; surgical department, Edward M. Dooley, Vertner Kenerson, William J. O'Donnell; alternates, Frederick M. Boyle, Edward H. Brady, Daniel Murphy; department of gynecology, S. Y. Howell; alternates, Cornelius C. Carr and M. A. Sullivan; department of electrotherapeutics, J. H. Daniels, P. H. Hourigan; special diagnosis, Thomas B. Carpenter: laryngologist, W. S. Renner; department of ophthalmology, Robert K. Grove; genitourinary surgeon, J. Henry Dowd; department of neurology, William C. Krauss; department of dermatology, A. E. Diehl.

The consulting staff consists of R. L. Banta and William C. Callanan, physicians, and Eugene A. Smith and Francis J. Carr, surgeons.

---

THE Niagara Hospital, located at 261 Georgia street, Buffalo, is an incorporated institution, owned and managed by physicians, and is open for the reception of surgical, medical and maternity cases. Physicians have the privilege of treating their ward cases. A training school for nurses has been established. Dr. Francis W. McGuire is president of the institution and Dr. Francis M. O'Gorman is the secretary.

---

SAINT LUKE'S HOSPITAL in the city of Utica, which for the past twenty years has been a private hospital of fifty beds, is to have a new building on a new and better site, the gift of Mr. and Mrs. Frederick T. Proctor, of Utica. The new building, which is now nearing completion, is to have eighty beds, and is a fireproof structure of brick, stone, and steel, of the most approved modern type. The entire expense of the building and furnishing is borne by this one family, and the structure completed is to be turned over to the present board of trustees of Saint Luke's Home and Hospital. This is the largest single gift that has ever been made for philanthropic and charitable purposes in that part of the state.

---

THE New York Skin and Cancer Hospital, Second avenue, corner Nineteenth street, through its governors announces that Dr. L. Duncan Bulkley will give a sixth series of clinical lectures on diseases of the skin, in the Out-patient Hall of the hospital, on Wednesday afternoons, commencing November 2, 1904, at 4.15 o'clock. The course will be free to the medical profession.

---

DR. M. A. CROCKETT, adjunct professor of obstetrics and clinical gynecology, Buffalo University, has been granted a year's leave of absence and will spend the winter at Pinehurst, N. C. Early in the spring Dr. and Mrs. Crockett will start on a foreign tour and will spend the summer traveling on the continent.

---

DR. REGINA FLOOD-KEYES, clinical instructor in obstetrics and diseases of women at Buffalo University, will occupy Dr. Crockett's teaching position during his absence.

